# Prevalence and influencing factors of chronic post-traumatic stress disorder in patients with myocardial infarction, transient ischemic attack (TIA) and stroke – an exploratory, descriptive study

**DOI:** 10.1186/s12888-021-03303-1

**Published:** 2021-06-07

**Authors:** Aurora Dollenberg, Sebastian Moeller, Caroline Lücke, Ruihao Wang, Alexandra P. Lam, Alexandra Philipsen, Jürgen M. Gschossmann, Falk Hoffmann, Helge H. O. Müller

**Affiliations:** 1 School of Medicine and Health Sciences, Medical Campus, University of Oldenburg, Oldenburg, Germany; 2 Faculty of Health/School of Medicine, Integrative Psychiatry and Psychotherapy, Witten/Herdecke University, Witten, Germany; 3 Universitätsklinikum Bonn AöR, Klinik und Poliklinik für Psychiatrie, Bonn, Germany; 4 Universitätsklinikum Erlangen, Klinik und Poliklinik für Neurologie, Erlangen, Germany; 5 Friedrich-Alexander Universität Erlangen-Nürnberg, Erlangen, Germany; 6 Klinikum Forchheim-Fränkische Schweiz gGmbH, Forchheim, Germany; 7 Division of Medical Psychology, Universitätsklinikum Bonn, Bonn, Germany; 8 Abteilung für Psychiatrie und Psychotherapie, Lehrstuhl für integrative Psychiatrie und Psychotherapie Private Universität Witten/Herdecke Gemeinschaftskrankenhaus Herdecke gGmbH, Gerhard-Kienle-Weg 4, 58313 Herdecke, Germany

**Keywords:** PTSD, TIA, Stroke, Myocardial infarction, Depression, Anxiety, Coping strategies, HRQoL

## Abstract

**Background:**

Cardio- and cerebrovascular events such as myocardial infarction (MI), stroke and transient ischemic attack (TIA) are leading causes of death and disability and have also been associated with poor mental outcomes. In addition, cardio- and cerebrovascular events may pose the risk of experiencing a sudden traumatic occurrence of symptoms during ictus and thus contribute to high rates of PTSD as well as high rates of subsequent depression and anxiety. Moreover, MI, TIA and stroke survivors with PTSD, depressive and anxiety symptoms may have poorer health-related quality of life (HRQoL) and poorer disease prognosis than patients who do not develop psychiatric symptoms after ictus. However, data on the prevalence of PTSD, anxiety and depression, as well as the HRQoL, coping strategies and potential risk factors for development of PTSD in these patients, are rare.

**Methods:**

In an exploratory, descriptive study we interviewed 112 patients (54 MI, 18 TIA, 40 stroke; mean age: 69.5 years, 55.4% males) from three general physician practices and used psychometric self-assessment tools to determine the occurrence of PTSD and psychosomatic comorbidity, anxiety and depression and to assess HRQoL and coping strategies. We evaluated disease severity and compared the patient groups to each other. Moreover, we assessed psychological outcome differences between patients with or without PTSD after ictus.

**Results:**

The prevalence of PTSD after MI, TIA and stroke was 23.2%. The patients who developed PTSD had higher rates of depression, anxiety and maladaptive coping as well as reduced HRQoL. Adaptive coping was positively related to better mental HRQoL and negatively related to anxiety and depression. Disease severity of MI, TIA and stroke was not related to PTSD, depression, anxiety or physical HRQoL.

**Conclusions:**

Experiencing MI, TIA or stroke means confronting a life-threatening event for those affected and, therefore, these can be regarded as traumatic events. Cerebral and cardiovascular events increase the risk of developing chronic PTSD with subsequent increased depression and anxiety and reduced HRQoL. These findings emphasize the need for early screening and diagnosis of PTSD in somatically ill patients, which should be followed by specialized treatment, as PTSD hampers overall (somatic) disease prognosis.

**Trial registration:**

German Clinical Trials Register, DRKS00021730, https://www.drks.de/drks_web/navigate.do?navigationId=trial.HTML&TRIAL_ID=DRKS00021730, registered 05/19/2020 - Retrospectively registered.

## Background

Cardio- and cerebrovascular events such as myocardial infarction (MI), stroke and transient ischemic attack (TIA) are leading causes of death and disability [1]. All conditions have been associated with poor mental outcomes, e.g., depression and anxiety, and worse quality of life. Post-stroke depression (PSD) can be ascribed as the most prevalent psychiatric complication of acute stroke and TIA [2, 3]. PSD affects about 30% of patients within a five-year period after stroke or TIA [2, 4, 5]. Moreover, MI, TIA and stroke are described by a dramatic and sudden onset. Thus, all conditions may pose the risk of experiencing a sudden traumatic occurrence of symptoms during ictus. This experience is likely to contribute to higher rates of PTSD and also higher rates of subsequent depression and anxiety [6] than in the general population. In 1994, for the first time, the DSM-IV added life-threatening diseases such as MI, stroke and TIA as possible triggers of PTSD.

Various studies have shown that MI can induce PTSD and PTSD-like symptoms [7, 8]. Moreover, MI-induced PTSD may contribute to an increase in the overall mortality rate and rate of hospital stays in MI survivors [9]. According to Edmondson et al., PTSD is associated with a 2-fold increased risk of reinfarction or death [9, 10]. In addition, MI survivors with PTSD report a significantly poorer health-related quality of life (HRQoL) [11]. Moreover, PTSD is associated with reduced drug adherence [12] and may subsequently deteriorate disease prognosis. Young age, female sex, a psychiatric history and maladaptive coping strategies may be risk factors for developing PTSD after MI [13, 14].

Additionally, in stroke patients, residual impairment and sequelae of permanent neurological deficits have an impact on HRQoL and may therefore contribute to psychological and psychosocial problems [15, 16]. Thus, increased prevalence of PTSD, depression and anxiety are found in stroke survivors [15, 17, 18]. Similar to patients with MI, younger age, a medical history including several strokes or TIAs and physical impairment due to stroke are risk factors for the development of PTSD after ictus [15, 19]. Social support seems to have a high protective value. Similar to cardiac events, deterioration in mental and physical HRQoL was observed in stroke survivors with PTSD [20]. Compared to patients with PTSD after MI [21], PTSD patients after stroke or TIA may have a poorer prognosis. If not adequately treated, PTSD symptoms tend to become chronic and persist for several years [6, 21]. According to the “National Comorbidity Survey”, approximately 1/3 of affected patients show a significant decrease in symptoms after one year. In more than 1/3, specific symptoms were found several times a week even 10 years after the event. If no adequate treatment occurred, PTSD symptoms were present for approximately five years, while symptoms persisted for approximately three years in patients that received symptom treatment [22]. Although MI, stroke and TIA are highly prevalent [1, 23], only a few studies have assessed the prevalence of PTSD, anxiety and depression in these patient cohorts [24–27]. Moreover, data on coping strategies in these patients are even rarer [27–29].

Therefore, in an exploratory, descriptive study we aimed to describe the prevalence of PTSD, depression, anxiety, HRQoL and coping styles in patients who have experienced MI, stroke or TIA and compare the patient groups to each other. Moreover, we attempted to identify psychological outcome differences between patients with or without PTSD after ictus.

## Methods

In an exploratory, descriptive study from April 1, 2017, to October 2018, 112 patients who had survived MI, stroke or TIA were interviewed. The study took place in urban practices in Wolfenbüttel, Braunschweig and Schladen, Germany. The study was approved by the Ethics Committee of the University of Oldenburg (169/2016) and performed in accordance with the ethical standards as set forth in the Declaration of Helsinki and its later amendments. The study is registered in the German Clinical Trials Register (DRKS00021730). Adult subjects who were diagnosed with one of the above somatic diagnoses at least three months and at most five years ago were eligible for inclusion. Non-German speakers were excluded from participating in the study. Only patients who did not have a psychiatric disorder, e.g., depression, anxiety disorder, PTSD or psychosis, before ictus were included in the study. All patients who fulfilled the inclusion criteria were informed about the study by an experienced interviewer (AD) during the general practitioner’s consultation. All subjects gave their written informed consent prior to their voluntary participation in the study. All research participants were informed about their right to withdraw from the study at any time and without giving any reason. The survey was performed in a structured expert-guided interview. Each interview lasted approximately 2 h on average. To avoid rater bias, the interview was performed by a single rater (AD). The questionnaires included the following validated psychometric instruments: *PC-PTSD* (Primary Care Checklist for PTSD), *CAPS-5* (Clinician-Administered PTSD Scale for DSM-5), *Brief COPE* (Coping Strategies Inventory), *HADS* (Hospital Anxiety and Depression Scale), *SF-12* (Short Form 12 Health Survey) and a sociodemographic questionnaire. If the *PC-PTSD* screening test was positive, another detailed survey (*CAPS-5*) followed to ensure the PTSD diagnosis. The GRACE score (Global Registry of Acute Coronary Events Score) was filled out retrospectively based on the patient data from the medical discharge reports. The mNIHSS (Modified National Institutes of Health Stroke Scale) was scored based on the results of the current neurological examinations in practice.

The following questionnaires and scoring systems were used:

### Primary care-PTSD screen (PC-PTSD)

The German version of the Primary Care-PTSD Screen (*PC-PTSD*) was used to assess the occurrence of PTSD symptoms. The *PC-PTSD* is a validated brief 4-item instrument developed by the Veteran’s Administration to screen for PTSD in primary care settings [30]. The PC-PTSD consists of four items that broadly correspond to the DSM--IV diagnostic criteria: intrusion; avoidance, hyperarousal and numbness or detachment (from others, activities, or surroundings). Those who endorsed at least 3 items screened positive on this scale. We further investigated patients who were screened positively *PC-PTSD* by asking them to subsequently complete the *CAPS-5*.

### Clinically administered PTSD scale (CAPS-5)

*The CAPS-5* is a structured clinical interview that covers the 20 symptoms of PTSD described in the DSM-5 [31]. The *CAPS-5* is a 30-item questionnaire [32]. Furthermore, overall symptom severity as well as global, social, occupational and personal impairment are assessed. The main criterion (A), the traumatic event, is assessed with the added Life Events Checklist for the DSM-5 (*LED-5*). First, the subject was read a list of questions about their mental condition after the serious illness, e.g., MI, then the frequency of symptoms was rated on a four-point scale and intensity of symptoms was rated on one five-point scale. The examiner assessed the validity of the statements, the overall severity of the symptoms and the improvement in symptoms upon repeated assessment or the improvement in symptoms within the past six months. The diagnosis of PTSD was established by dichotomizing the individual symptoms as “present” or “not present”, according to the DSM-5 diagnostic rules. A symptom was only considered to be present if the corresponding frequency score was rated with at least 1 point (cut-off value) and the intensity score was rated with at least 2 points (cut-off value). Dissociative phenomena were assessed as positive in the *CAPS-5* if the score was more than 4 points for the sum of frequency and intensity for either the “depersonalization” or “derealization” symptoms [33].

### Short form (SF)-12

HRQoL can be understood as a complex concept encompassing social, psychological, and physical aspects of wellbeing and functioning. HRQoL was assessed using the German version of the Short Form *(SF)-12* questionnaire [34]. The *SF-12* uses only 12 of the 36 questions from the SF-36, and the 12 questions can be abstracted from the answers provided in the *SF-36* [35]. The questions of the SF-12 were evaluated as follows: each given answer option in the SF-12 questionnaire was assigned a numerical value from one to a maximum of six. Questions 1, 8, 9 and 10 of the SF-12 were reversed so that high values ​​always corresponded to a better state of health. The 12 items are aggregated into two health summary scales that reflect physical (PCS) and mental (MCS) components ranging from 0 (worst) to 100 (best). The component scores for the *SF-12* are norm-based, with a mean score of 50 and a standard deviation of ±10. A higher score reflects better HRQoL [34].

### Hospital anxiety and depression scale (HADS)

To assess the prevalence of depressive and/or anxiety symptoms in the past week, we used the HADS [36]. The HADS is a well validated screening instrument for psychological burden in patients with somatic diseases and is widely used. It consists of two subscales (anxiety and depression) comprising 7 items each. Items are scored on a four-point Likert scale between 0 and 3. Subscale scores of 7 and below are considered as having no depressive or anxiety symptoms, whereas scores between 8 and 10 are considered borderline, and scores of 11 and above indicate clinical manifestations of depressive or anxiety symptoms [36, 37].

### Coping strategies inventory (brief COPE)

The *COPE* inventory, which contains 12 subscales, was applied to assess the patient’s disposition for using maladaptive/adaptive coping strategies [38]. It consists of 28 items scored on a four-point Likert scale, where 1 means “not at all”, 2 means “some”, 3 means “quite often”, and 4 means “a lot”. The sum of maladaptive coping strategy scores (denial, substance use, behavioral disengagement, self-distraction, self-blame) was assessed as an indicator of maladaptive coping. The sum of the adaptive coping strategy scores (active coping, emotional support, expression of emotions, instrumental support, positive reinterpretation, planning, humor and acceptance) was assessed as an indicator of adaptive coping. The higher the maladaptive or adaptive coping test score, the more patients habitually use maladaptive or adaptive coping strategies, respectively [38].

### Global registry of acute coronary events (GRACE) score

To assess the severity of MI on admission (index event), the *GRACE* score was used [39]. The *GRACE* score has been extensively validated. It is a strong predictor of inpatient mortality and undesirable outpatient outcome parameters, e.g., PTSD in MI patients. One of the parameters is the so-called Killip classification, which is based on the clinical signs of heart failure. Other parameters of the *GRACE* score include patient age, heart rate, systolic blood pressure, serum creatinine level, cardiac arrest at hospital entry, ST segment deviation on the ECG and elevated cardiac markers. The severity of MI was divided into three possible levels depending on the *GRACE* score: mild with less than 100 points (inpatient mortality < 1%), moderate with 101–170 points (inpatient mortality 1–9%) and severe with 171–250 points (inpatient mortality> 9%) [40]. The higher the *GRACE* score, the more severe the MI [39].

### Modified National Institutes of Health stroke scale (mNIHSS)

The *mNIHSS* is a scoring system that enables a quantitative assessment of the severity of TIA and stroke in outpatients at the time of the survey. The *mNIHSS* comprises 11 items that assess state of consciousness, eye movement, visual field, facial muscles, movement of the extremities, coordination of the extremities, sensitivity, language production and understanding, speech function and attention [41]. Each of the 11 items in the *mNIHSS* is assigned a numerical value from 0 to a maximum of 4 according to the results of the neurological examinations. An *mNIHSS* score is then determined by summing the item scores. The severity of the neurological deficits was divided into three possible levels: no-mild deficits at 0–4 points, moderate deficits at 5–20 points and severe deficits at 21–31 points. The higher the score on the *mNIHSS*, the more extensive and severe the stroke [41].

### Sociodemographic data

To assess the sociodemographic data, age, sex, marital status, number of children, education and vocational training and employment were determined. These data were collected using selected questions from the DEGS98 (Federal Health Survey 1998) [42].

### Statistics

This study was a cross-sectional exploratory study. For the descriptive statistical evaluation, nominally scaled or categorical characteristic variables are shown as frequencies and percents. Continuous variables were presented by specifying mean values ​​and associated standard deviations. Most of the calculations were carried out using continuous data (e.g., correlative calculations), including those of the psychometric survey instruments used. Nominal-scaled and continuous variables were evaluated using the χ^2^ test or Fisher exact test as appropriate and analysis of variance (ANOVA), respectively. Comparisons of the mean values ​​of two unpaired samples were carried out with the t-test to determine differences between the study groups (MI, TIA and stroke). All significance tests were two-sided. A commercially available statistical program (SPSS™ Version 25, SPSS Inc., Chicago, Ill, USA) was used for data analysis. Significance was consequently set at *p* ≤ 0.05.

## Results

The total sample consisted of 50 women (44.6%) and 62 men (55.4%). The mean age of the female patients was higher (73.9 ± 17.1 years) than that of the male patients (66.0 ± 12.2 years, *p* = 0.006). Table 1 summarizes the participants’ sociodemographic information (Table 1). 54 patients with MI, 18 patients with TIA and 40 patients with stroke were included in the study.

**Table 1:** Descriptive sample characteristics and clinical characteristics in 54 patients who have experienced a myocardial infarction (MI), 18 patients who have experienced a transient ischemic attack (TIA) and 40 patients who have experienced a stroke

		MI *N* = 54 (48.2%)	TIA *N* = 18 (16.1%)	Stroke *N* = 40 (35.7%)	*p*-value
Age ± SD		68.1 ± 13.7	72.7 ± 18.7	70.0 ± 15.2	0.532
Sex	female	18 (33.3%)	11 (61.1%)	21 (52.5%)	0.056*
	male	36 (66.7%)	7 (38.9%)	19 (47.5%)	
Marital status	single	4 (7.4%)	3 (16.7%)	2 (5.0%)	**0.423**^**†**^
	within a partnership	38 (70.4%)	9 (50.0%)	26 (65.0%)	
	married	12 (22.2%)	6 (33.3%)	12 (30.0%)	
Number of children	0	8 (14.8%)	4 (22.2%)	5 (12.5%)	0.443^†^
	1	10 (18.5%)	4 (22.2%)	9 (22.5%)	
	2	28 (51.8%)	7 (38.8%)	16 (40.0%)	
	3+	8 (14.9%)	3 (16.8%)	10 (25.0%)	
Graduation/Education	basic prim. School	48 (88.9%)	15 (83.3%)	35 (87.5%)	0.238^†^
	high school / uni ent	4 (7.4%)	1 (5.6%)	2 (5.0%)	
	without	2 (3.7%)	2 (11.1%)	3 (7.5%)	
Professional qualification	apprenticeship	34 (63.0%)	13 (72.2%)	27 (67.5%)	0.082^†^
	craftsman	2 (3.7%)	2 (11.1%)	0 (0.0%)	
	university	5 (9.3%)	0 (0.0%)	0 (0.0%)	
	none	13 (24.1%)	3 (16.7%)	13 (32.5%)	
Occupation	employee	51 (94.4%)	17 (94.4%)	40 (100.0%)	0.264^†^
	self-employed	1 (1.9%)	1 (5.6%)	0 (0.0%)	
	none	2 (3.7%)	0 (0.0%)	0 (0.0%)	
Disease severity	GRACE (mean ± SD) mNIHSS (mean ± SD)	125.2 ± 36.8	0.4 ± 0.9	7.6 ± 3.4	< 0.001
PTSD	PTSD+ (n = 26)	7 (13.0%)	3 (16.7%)	16 (40.0%)	0.007^†^
	PTSD- (n = 86)	47 (87.0%)	15 (83.3%)	24 (60%)	
HADS-D	mean ± SD	6.0 ± 4.6	9.3 ± 5.2	10.4 ± 5.0	< 0.001
HADS-A	mean ± SD	6.3 ± 3.1	7.6 ± 2.9	8.4 ± 4.4	0.022

basic prim. School: basic primary school. Uni entr: University entry diploma. MI: myocardial infarction. TIA: transient ischemic attack. GRACE: Global Registry of Acute Coronary Events Score. mNIHSS: Modified National Institutes of Health Stroke Scale. SD: Standard Deviation. PTSD: Post-traumatic stress disorder. HADS: Hospital Anxiety and Depression Scale, D-depression subscale, A- anxiety subscale. *- Chi-squared Test, ^†^- Fisher’s exact test

### PTSD

All patients answered the PC-PTSD scale of whom 28 screened positive. These 28 participants completed the CAPS-5. A total of 23.2% (*n* = 26) of the 112 study participants fulfilled the criteria for PTSD. The highest PTSD prevalence was found in the stroke patients (40.0%, *n* = 16), followed by the TIA patients (16.7%, *n* = 3) and the MI patients (only 13.0%, *n* = 7, *p* = 0.007). There was an average disease severity of 125.2 ± 36.8 (moderate) in MI patients. TIA patients had only mild disease severity (0.4 ± 0.9), which differed significantly from stroke patients who had moderate disease severity (7.57 ± 3.4, *p* < 0.001). Disease severity in MI patients did not differ significantly between those with and without PTSD (117.1 ± 28.4 vs. 126.4 ± 38.1, *p* = 0.540). However, in stroke patients, disease severity in patients with PTSD was significantly higher than that in stroke patients without PTSD (8.0 ± 4.5 vs. 4.0 ± 3.7, *p* = 0.001).

### HADS

The average *HADS* score for depression was lowest in MI patients (6.0 ± 4.6), higher in TIA patients (9.3 ± 5.2) and highest in stroke patients (10.4 ± 5.0, *p* < 0.001). The average *HADS* score for anxiety was again lowest in MI patients (6.3 ± 3.1), higher in TIA patients (7.6 ± 2.9) and highest in stroke patients (8.4 ± 4.4, *p* = 0.022). Of the 26 PTSD patients with MI, stroke or TIA, 52.8% (*n* = 19) had a *HADS* depression score of more than eleven, while 47.2% (*n* = 17) had a *HADS* depression score value of 11 points or lower. Of the 86 PTSD-negative patients with MI, stroke or TIA, only 18,4% (*n* = 14) had a HADS depression score of more than eleven, while 81,6% (*n* = 62) had a HADS depression score value of 11 points or lower (*p* < 0.001). The lowest number of patients with a HADS depression score of more than 11 points was in the MI patients (13%, *n* = 7), while there were more in TIA patients (33.3%, n = 6) and the highest number in stroke patients (50%, *n* = 20, p < 0.001).

Of the 26 PTSD patients with MI, stroke or TIA, 50% (*n* = 13) had a HADS anxiety score of more than eleven, while 50% (n = 13) had a HADS anxiety score of 11 points or lower. Of the 86 PTSD-negative patients with MI, stroke or TIA, only 11,6% (*n* = 11) had a *HADS* anxiety score of more than eleven, while 88,4% (*n* = 76) had a HADS anxiety score value of 11 points or lower (*p* < 0.001). The number of patients with a HADS anxiety score of more than eleven was similar between the groups (MI patients: 14.8%, *n* = 8; TIA patients: 16.7%, *n* = 3; stroke patients: 30%, *n* = 12, *p* = 0.179).

### Dissociation

The prevalence of dissociation was 3.6% in the whole patient population. Only patients with PTSD had dissociated (*p* = 0.002).

### Coping in patients with MI, TIA and Stroke (COPE-A, COPE-M)

Only adaptive coping had significant effects on PTSD, depression and anxiety. PTSD patients (*n* = 26) had significantly lower COPE-A mean values (35.7 ± 5.2) than PTSD-negative (*n* = 86) patients (39.5 ± 6.2, *p* = 0.005). Depressive patients with a *HADS* depression score value of more than eleven (*n* = 33) had significantly lower *COPE-A* mean values (35.6 ± 5.1) than had patients (*n* = 79) without depression (40.0 ± 6.2, p < 0.001). Anxious patients with a *HADS* anxiety score value of more than eleven (*n* = 23) had significantly lower *COPE-A* mean values (36.2 ± 4.8) than had patients (*n* = 89) without anxiety (39.4 ± 6.4, *p* = 0.012). The mean values ​​of the *COPE-A* and *COPE-M* did not significantly differ between the MI, stroke and TIA patients (*p* > 0.05).

### Quality of life in patients with MI, stroke or TIA (SF-12)

*The* PCS and MCS of all study participants were numerically below the reference values ​​of the German general population [43] (Table 2).

**Table 2:** PCS and MCS of all study participants in comparison to the German general population

mean ± SD	MI N = 54	TIA N = 18	Stroke N = 40	p-value	General Population
MCS	41.7 ± 10.2	36.9 ± 10.4	37.8 ± 8.3	0.072	51.4 ± 0.30
PCS	44.6 ± 8.7	39.6 ± 6.6	38.3 ± 7.6	0.001	49.3 ± 0.30

MCS: mental component summary (of the SF-12), PCS: physical component summary (of the SF-12), MI: myocardial infarction, TIA: transient ischemic attack

MCS was highest in MI patients (44.6 ± 8.7), second highest in TIA patients (39.6 ± 6.6) and lowest in stroke patients (38.3 ± 7.6, p = 0,001). The mean values of the PCS were similar between the three groups (*p* = 0.072).

MCS was significantly lower in patients (*n* = 26) with PTSD (37.0 ± 6.9) than in patients (*n* = 86) without PTSD (42.9 ± 8.5, *p* = 0.002). PCS was again significantly lower in patients with PTSD (36.8 ± 7.3) than in patients without PTSD (40.4 ± 10.3, *p* = 0.004). The MCS, but not in the PCS, was significantly different (*p* = 0.021) between the three groups (Table 3).

**Table 3:** MCS comparison in patients with MI, TIA and stroke

MCS	Below average	Average	Above average	p-value
MI	7.4%	63%	29.6%	0.021
TIA	11.1%	83.3%	5.6%	
**S**troke	22.5%	67.5%	10%	

MCS: mental component summary (of the SF-12), MI: myocardial infarction, TIA: transient ischemic attack

## Discussion

Diagnosis of MI, TIA or stroke means confrontation with a life-threatening event for those affected and can therefore be regarded as a traumatic event. Cerebral and cardiovascular events may increase the risk of developing chronic PTSD. There has been an abundance of evidence, particularly in recent years, demonstrating the enormous influence of psychological factors not only on HRQoL but also on the course of the disease and the survival chances of patients with MI, TIA or stroke [44–46]. These concepts and the corresponding therapeutic approaches are hardly known in the routine medical care of these patients. In addition to medical treatment, a particular challenge is the early detection of high-risk patients who may develop symptoms of PTSD in the future. Only early detection of these symptoms enables fast and effective therapy. This is the only way to prevent further chronification, therapy delay and prolongation of the length of hospital stay followed by a poorer prognosis.

In our study, the prevalence of chronic PTSD in MI, TIA and stroke patients was 23.2%, which is more than 4 to 40 times higher than the lifetime prevalence in the general population in Europe (0.56 to 6.67%) [47]. This indicates an outstanding high-risk population for PTSD hampering their overall prognosis. Our findings highly emphasize the need for an early screening and diagnosis of PTSD in cardiovascular and neurovascular patients, which should be followed by a specialized treatment of any identified PTSD symptoms. The sociodemographic characteristics of our patients, e.g., age, family status, number of children, level of education or occupation of the sample, were not associated with the PTSD prevalence estimates. Moreover, we did not find significant statistical relationships between the severity of the disease, i.e., MI, TIA or stroke, and the development of chronic PTSD. Numerous studies did not find correlations between disease severity and later manifestations of PTSD symptoms [48–50]. However, structured prospective examinations with larger cohorts should be conducted in the future. The prevalence of depression in MI, TIA and stroke patients was 29.5%, which is more than twice as high as in the general population (2–12%) [51, 52].

In the comparisons between the groups, we observed highly significant differences. The prevalence of depression was 13.0% in MI patients, 33.3% in TIA patients and 50.0% in stroke patients. Previous studies have shown similarly increased depression rates after stroke (3–39%) [53–55]. This is probably because the diagnostic symptoms of depression and stroke overlap in the DSM-5 [56], and our sample consisted mainly of patients with moderate stroke severity. Stroke severity and physical disability are the most consistent predictors of poststroke depression [54, 57]. In contrast to anxiety disorder, depression may increase the mortality and disability of stroke patients [58]. Probably due to the increased depression rate, the mental and physical HRQoL of our patient groups were significantly lower than the quality of life of the general population. This is confirmed by the results of other studies on HRQoL and depression in adults [59]. In addition, the results of our study are in line with previous findings that depression is associated with increased comorbidity [47, 60]. More than half (52.8%) of our PTSD-positive patients showed depressive symptoms. This is again in line with results from the literature, according to which about half of the patients with PTSD also suffer from depression [61, 62]. The prevalence of anxiety was 20.5% in the entire patient cohort, which is more than two times higher than that in the general population [63]. However, the anxiety prevalence did not differ between the groups. Consistent with the literature [5, 64–66], concurrent depression was common in patients with anxiety, which again confirmed the need to treat depression in people with anxiety after MI, TIA and stroke. Half of our PTSD-positive patients had anxiety symptoms. This is not surprising, as PTSD appears to be a common comorbidity of anxiety disorder [58, 67]. Our three patient groups developed adaptive and maladaptive coping after MI, TIA and stroke equally. Multiple comparisons of the three patient groups did not show any significant differences between the coping strategies. In the literature, most negative life events appear to elicit both types of coping strategies [68]. The maladaptive coping style did not predict the development of PTSD, which is in contradiction to the few results in other studies [69]. In our patients, we observed significant differences in adaptive coping only between those who additionally had anxiety, depression or PTSD and patients who did not develop these symptoms. Consistent with other studies [45, 70], we found that the more our patients used adaptive coping strategies, the higher the mental HRQoL they were able to achieve, and this association was significant. In addition, patients with a higher depression score showed both significantly less adaptive coping ability and significantly more maladaptive coping ability. This finding is supported by numerous studies [71–73]. Nevertheless, regulatory and coping strategies are not consistently beneficial or maladaptive in response to stressful events [74]. There is evidence that some of the coping strategies can be both adaptive and maladaptive [75, 76]. In patients with chronic life stressors Waugh et al. found that positive distraction was related to higher well-being and positive emotions, and fewer depressive symptoms especially when controlling for avoidance. Thus, positive distraction can be an adaptive disengagement coping strategy for chronic stressors [75]. Wolgast and Lundh suggest that distraction may be either adaptive or maladaptive, depending on whether it is combined with an attitude of acceptance or avoidance [76].

Our findings need to be interpreted cautiously due to the overall small sample size. Particularly the low number of TIA patients makes it difficult to make inferences about the minority of patients in this small group who have elevated PTSD symptoms. Our findings should be further evaluated in larger prospective studies. Thus, the comparison of three different patient groups up to five years after ictus can only provide limited findings. Due to the study design severe cases of stroke and MI might be underrepresented. Moreover, the cross-sectional design is a limitation of our study. A prospective rather than a cross-sectional study design would require to assess changes in coping styles and PTSD symptoms over time. Additionally, there are differences in DSM-IV and DSM-5 regarding medical events that may be considered traumatic. These differences have to be taken into account when interpreting our findings on PTSD. Another limitation of our study is that the exact time between the health event (i.e., stroke, TIA, MI) and the time of the survey was not recorded. The length of time between ictus and interview could have an influence on the results. Still, we were able to examine an unselected outpatient population with a comprehensive clinical and neuropsychological assessment.

## Conclusion

To conclude, the prevalence of chronic PTSD in MI, TIA and stroke patients was 23.2%. The experience of an MI, a TIA or a stroke increased the risk of developing chronic PTSD and associated depression, anxiety, maladaptive coping and reduced HRQoL. We found that adaptive coping was positively related to mental HRQoL and negatively related to anxiety and depression. By contrast, variables such as age, sex, marital status, number of children, level of education, duration of illness and maladaptive coping do not seem to play a role in any of our patient cohorts. The severity of the somatic disease was not related to the development of PTSD, depression, anxiety or physical HRQoL.

Physicians should have comprehensive knowledge of the frequency and risk factors of chronic PTSD to identify PTSD symptoms at an early stage after MI, TIA or stroke.

Based on our recent findings, we recommend a structured and standardized screening for PTSD and PTSD-like symptoms in the named high-risk groups, which, in ideal circumstances, should start in parallel with somatic treatment and be followed by specific psychological treatments as needed.

## Data Availability

The datasets generated during and analyzed during the current study are available from the corresponding author on reasonable request.

## Abbreviations

TIA: transient ischemic attack
MI: myocardial infarction
PTSD: post-traumatic stress disorder
ICD: International Classification of Diseases
HRQoL: health-related quality of life
DSM-5: Diagnostic and Statistical Manual of Mental Disorders fifth edition
e.g.: exempli gratia, for example
PC-PTSD: Primary Care Checklist for post-traumatic stress disorder
CAPS-5: Clinician-Administered post-traumatic stress disorder Scale for Diagnostic and Statistical Manual of Mental Disorders fifth edition
COPE: Coping Strategies Inventory
HADS: Hospital Anxiety and Depression Scale
SF-12: Short Form 12 Health Survey
GRACE score: Global Registry of Acute Coronary Events Score
mNIHSS: Modified National Institutes of Health Stroke Scale
DEGS98: German health interview and examination survey for adults
ANOVA: analysis of variance
SPSS: Statistical Package for the Social Sciences
COPE-A: adaptive coping
COPE-M: maladaptive coping.
PCS: physical component summary
MCS: mental component summary
